# The Role of Periarticular Knee Muscle Torques in Ensuring the Body Balance of Older Adults with Balance Disturbances

**DOI:** 10.3390/jcm14093251

**Published:** 2025-05-07

**Authors:** Piotr Prochor, Łukasz Magnuszewski, Paulina Zalewska, Michał Świętek, Zyta Beata Wojszel, Szczepan Piszczatowski

**Affiliations:** 1 Department of Biomaterials and Medical Devices Engineering, Institute of Biomedical Engineering, Faculty of Mechanical Engineering, Bialystok University of Technology, 15-351 Bialystok, Poland; paulina.zalewska@sd.pb.edu.pl (P.Z.); s.piszczatowski@pb.edu.pl (S.P.); 2 Department of Geriatrics, Medical University of Bialystok, 15-089 Bialystok, Poland; magnuszewskilukas@gmail.com (Ł.M.); zyta.wojszel@umb.edu.pl (Z.B.W.); 3 Department of Otolaryngology, University Clinical Hospital in Bialystok, 15-276 Bialystok, Poland; michalswietek@wp.pl; 4 Department of Geriatrics and Internal Diseases, Hospital of the Ministry of Interior and Administration in Bialystok, 15-471 Bialystok, Poland

**Keywords:** balance disorders, knee joint, muscle torques, older people

## Abstract

**Background**: The role of the periarticular muscles of the knee joint in ensuring body balance is still ambiguous. Therefore, we conducted clinical and biomechanical assessments on 52 older adults (36 women and 16 men, age of 67.58 ± 7.30 years, body weight of 75.10 ± 13.42 kg, and height of 163.92 ± 8.80 cm) to determine the role of the knee muscles in balance maintenance. **Methods**: The clinical examination included the Dizziness Handicap Inventory (DHI), the Geriatric Depression Scale (GDS), the Performance-Oriented Mobility Assessment (POMA), the Functional Reach Test (FRT), the Falls Efficacy Scale—International (FES-I), and bioimpedance parameters (skeletal muscle mass—SMM—and its derived parameter—Diff SMM). The biomechanical assessment involved parameters that characterize muscle torques of knee joint extensors and flexors in isokinetic and isometric conditions, as well as changes in the centre of pressure (COP) position while standing with eyes open and closed. **Results**: Based on treatment history and DHI results (>10 points), 26 participants were identified as having balance disorders, while the remaining participants formed the control group. Statistical analysis was performed to determine differences between the groups. The groups significantly differed in terms of the results obtained from the DHI (*p* < 0.001) and GDS (*p* = 0.04) questionnaires as well as FES-I (*p* < 0.001) and POMA (*p* = 0.002) tests. While SMM (*p* = 0.012) was similar in the groups, Diff SMM (*p* = 0.04) significantly differed. The multiple regression analysis confirmed the knee joint extensor parameters’ significant role in predicting the COP path (*p* = 0.03 and *p* = 0.04 for two assumed models). **Conclusions**: The obtained results proved that the muscle torques of knee extensors can be used in the diagnostic process of older patients with balance disorders, indicating possible rehabilitation directions.

## 1. Introduction

An older person is an individual in the later stage of life, during which natural biological ageing processes lead to a progressive decline in the body’s functional reserves, decreased efficiency of organ systems, increased susceptibility to chronic diseases, and a slowdown in regenerative processes. Common features also include balance disorders, reduced muscle strength, and impaired motor coordination, which increase the risk of falls and limit independence in daily functioning [1].

According to the classification of the World Health Organization (WHO), older persons are defined as individuals aged 60 years and above. However, in certain social, cultural, and health programme contexts, the age of 55 years is also accepted as the lower threshold, particularly in populations with a lower average life expectancy [2].

Due to significant progress in medicine, the average human lifespan has significantly increased [3], directly related to the increasing proportion of older people in society. According to the World Health Organization (WHO), by 2050, individuals aged 60 and above will represent between 12% and 22% of the global population [4]. Although the COVID-19 pandemic temporarily disrupted this demographic shift [5], current trends indicate a steady return to pre-pandemic projections. As the ageing population grows, clinical practice is increasingly dominated by conditions typical of older adults—among which falls have become a critical and persistent concern. Falls, often resulting from balance disorders, are one of the most frequent and serious health issues affecting the elderly [6]. They not only pose an immediate risk of injury, such as fractures or head trauma, but also lead to complications including loss of independence, prolonged hospitalisations, and increased mortality. In everyday clinical care, managing fall-related incidents has become a routine, placing significant demands on healthcare professionals and systems. Preventing falls and addressing their consequences are now central priorities in geriatric care and rehabilitation planning. It is estimated that people over 65 are at a 15% risk of falling, increasing to 25% in people over 80 years of age [7].

The WHO defines a fall as “an event that results in a person coming to rest inadvertently on the ground or floor or other lower level” [8]. Several factors increase the risk of falls in older persons; among others, the following can be noted: medications, osteoarthritis, depression, dizziness, cerebellar damage, degenerative changes in the middle and inner ear, or muscle weakness [9,10,11]. However, there is a large proportion of falls of unknown origin, so there is a high need to improve and develop new diagnostic methods for balance disorders. Falls result in severe consequences for quality of life, such as fear of falling again [12], leading to physiological weakness [13], social isolation [14], or psychomotor issues [15], which all combined directly lead to the development of chronic diseases that can be avoided with regular physical activity [16]. In critical cases, falls lead to severe mechanical damage in bone tissues, especially hip joint fractures [17], and are a direct cause of premature mortality [18].

Currently, several questionnaires (such as the Dizziness Handicap Inventory—DHI—or the Geriatric Depression Scale—GDS) [19,20], functional tests (such as the Performance-Oriented Mobility Assessment—POMA—the Functional Reach Test—FRT—or the Falls Efficacy Scale—International—FES-I) [21,22,23,24], and body composition analysis (in particular to determine skeletal muscle mass—SMM) [25,26] are used to establish the potential risk of falls in older people. However, clinical reports suggest that the results obtained in studies mentioned above often do not allow a clear assignment of a patient to a risk group. For this reason, researchers are looking for other methods with which to potentially increase the quality of diagnosis of balance disorders in older adults.

One of the parameters used to describe body balance is movement of the centre of pressure (COP), which is usually evaluated on test platforms, and its excessive range can be directly used to distinguish fallers from non-fallers [27]. Currently, the available literature has extensive quantitative descriptions of the COP in older populations [28,29,30] concerning potential influencing factors, such as lower-extremity muscle forces, having a significant influence on this parameter. Despite some noticeable impact of muscle forces, especially abductors of the hip joint, on maintaining appropriate balance [31,32,33,34], the influence of the periarticular knee muscles remains ambiguous.

Therefore, the research aimed to assess the influence of knee joint muscle torques measured in isokinetic and isometric conditions on changes in COP movement and assess these parameters’ usefulness in diagnosing balance disorders in older people.

## 2. Materials and Methods

### 2.1. Study Design

A condensed overview of the study design is presented below in bullet points to enhance clarity and facilitate quick understanding of the methodological framework. This is used to summarize the core aspects of the study, including its structure, assessment tools, and analytical approaches. It highlights the most important procedures implemented throughout the study in a clear and logically ordered manner.

- Sample: 52 participants aged > 55 years.
- Recruitment: clinical and community sources in Bialystok, Poland.
- Assessments performed:
  - General: weight, height, and BMI.
  - Clinical: DHI, GDS, POMA, FRT, FES-I, and body composition (bioimpedance).
  - Biomechanical: muscle torques of knee flexors and extensors (isometric and isokinetic conditions), and centre of pressure (COP) position changes.
- Group allocation:
  - Balance disorders group: ≥ 1 fall in the past year and DHI ≥ 10.
  - Control group: no falls and DHI < 10.
- Statistical analysis:
  - Shapiro–Wilk and Levene’s tests.
  - Group comparisons: *t*-test or Mann–Whitney U.
  - Multiple regression to identify predictors of the COP path.

A comprehensive and detailed description of each procedure (including participant recruitment, group allocation, clinical as well as biomechanical assessments, and statistical approach) is provided in the subsequent section to ensure methodological transparency and reproducibility.

### 2.2. Detailed Description of Study Procedures

The study involved 52 people (36 women and 16 men) aged over 55, recruited from patients of the Department of Geriatrics of the Hospital of the Ministry of Interior in Bialystok, Poland, the Department of Otolaryngology, University Clinical Hospital in Bialystok, Poland, and from people associated with the University of the Third Age between 1 December 2022 and 30 September 2024. Recruitment was conducted through direct invitations during patient visits, referrals by attending physicians and physiotherapists, and through announcements distributed among senior community organizations. Interested individuals were initially screened for eligibility based on their age and general health status. Those who fulfilled the inclusion criteria were invited to participate, provided detailed information about the study procedures, and gave written informed consent. Participants were included in the study if they met the following criteria:

- Age of 55 years or older at the time of recruitment.
- Ability to ambulate independently without the use of mobility aids (e.g., walkers or wheelchairs).
- No history of acute orthopedic injuries or surgical interventions involving the lower limbs in the previous six months.
- No diagnosed neurological conditions significantly affecting balance (e.g., Parkinson’s disease, stroke, or multiple sclerosis).
- Consent to participate in clinical and biomechanical assessments.

The average age of the participants was 67.58 ± 7.30 years. Their mean body weight was 75.10 ± 13.42 kg, with an average height of 163.92 ± 8.80 cm. The resulting mean Body Mass Index (BMI) was 27.77 ± 3.88 kg/m². All participants signed a written consent form to participate in the research and underwent clinical evaluation (conducted by a physician and physiotherapist) and biomechanical parameter assessment (performed by biomedical engineers). The research was performed with the consent of the Bioethics Committee of the Medical University of Bialystok.

The clinical assessment concentrated on the potential impact of balance disturbances on a person’s quality of life, emotional health, risk of falls, and body composition analysis using the bioimpedance method. This assessment was used both for the classification of participants into the control and balance disorder groups (based primarily on DHI scores and fall history) and as key outcome measures. Their inclusion in the comparative analysis allowed for the evaluation of emotional-, functional-, and balance-related differences between the groups, as presented in the Results section. The clinical assessment included the following:

- The Dizziness Handicap Inventory (DHI) is a scale designed to measure the disability perceived by someone complaining of dizziness, vertigo, or unsteadiness. It is a 25-item self-report questionnaire that quantifies the impact of dizziness on daily life by measuring self-perceived handicaps in functional, emotional, and physical categories. The patient is asked to answer each question regarding dizziness or unsteadiness problems, explicitly considering their condition during the last month (No = 0; Sometimes = 2; and Yes = 4 points). Scores above 10 points should be referred to balance specialists for further evaluation [35].
- The 15-item Geriatric Depression Scale (GDS) is an instrument used to assess the possibility of depression in older adults. The patient is asked to answer each question explicitly considering his or her condition during the previous two weeks (0 or 1 point is assigned to the answer depending on the question). Scores above 5 points should be referred to more in-depth clinical evaluation for depression [36].
- The Performance-Oriented Mobility Assessment (POMA) is the first clinical balance assessment tool [37]. It measures an individual’s gait and balance abilities using 16 items of balance and gait. Each item is scored on a 3-point ordinal scale (0–2) with 28 possible points. The balance portion (POMA-B) contains nine items for a maximum score of 16 and the gait portion (POMA-G) includes seven items for a maximum score of 12. The test is reliable, valid, and responsive [38,39,40]. The POMA has excellent test–retest reliability in people with dementia [41].
- The Functional Reach Test (FRT) evaluates the forward stability of a standing subject who voluntarily extends one arm as far forward as possible in the horizontal plane, while keeping both heels in contact with the ground. The score is obtained by measuring the distance between the fingertip’s starting and end positions. The FRT’s good reliability and low inter-examiner variability have been demonstrated in various studies [42,43]. The FRT score correlates with the anteroposterior excursion of the centre of pressure (COP) [42,44]. Furthermore, the FRT also shows good reproducibility over time [42,45] and is sensitive to change [46,47]. Lastly, the FRT score is known to decrease with age, and an abnormally low score is a good predictor of the risk of falls [46].
- The Falls Efficacy Scale—International (FES-I), developed and validated by the Prevention of Falls Network Europe (ProFaNE), has become a widely accepted tool for assessing concern about falling [48,49]. Previous studies indicate that the FES-I has excellent reliability and validity across different cultures and languages [50]. The psychometric properties of the FES-I have been evaluated using classical test theory [48,49]. The original questionnaire contains 16 items scored on a four-point scale (1 = not at all concerned to 4 = very concerned). The shortened questionnaire contains seven items [48].
- Body composition assessment with a Jawon Medical X-Contact 357S (Jawon Medical Co., Ltd., Gyeongsan, Republic of Korea) bioimpedance analyzer was used to assess skeletal muscle mass (SMM) and the derived parameter—the difference between the obtained SMM value and its reference value (Diff SMM).

Based on medical history concerning falls in the previous year and the results of the DHI questionnaire, 26 persons (19 women and 7 men) were qualified to the group with balance disorders (DHI ≥ 10 points and a history of at least one fall in the previous year), and another 26 (17 women and nine men) to a control group (DHI ˂ 10 and no history of falls in the previous year).

The following parameters were determined within the biomechanical assessment:

- Muscle torques of knee flexors and extensors developed in isometric and isokinetic conditions.
- The centre of pressure (COP) position changes while standing with eyes open and closed.

**Figure 1:**
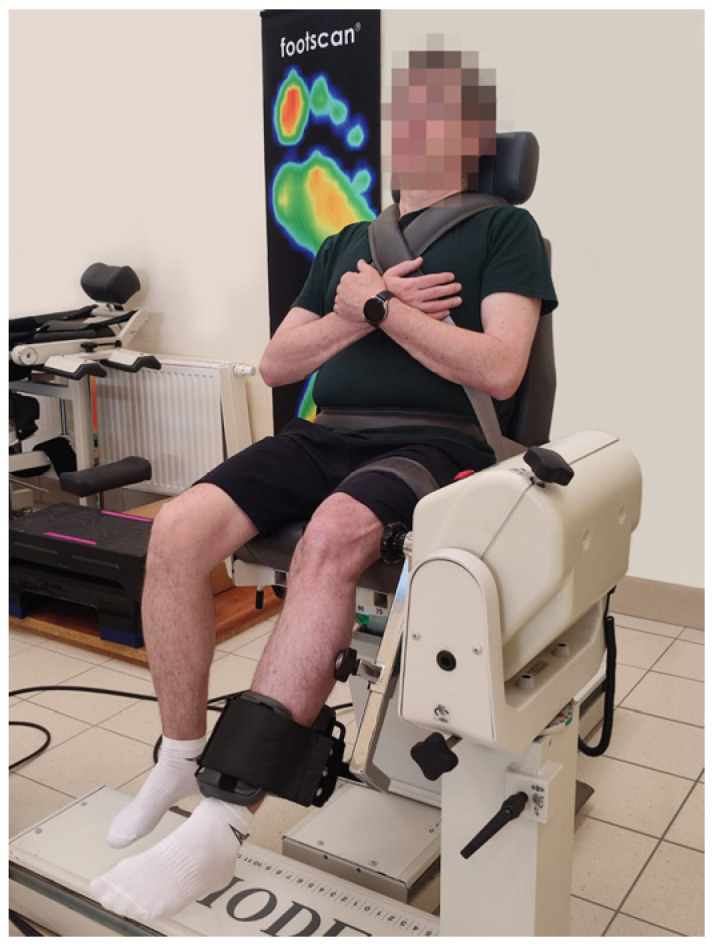
A patient undergoing assessment of the muscle torques of the knee flexors and extensors using the Biodex System 4 Pro dynamometer.

The muscle torques of the knee flexors and extensors were tested using a Biodex System 4 Pro dynamometer (Biodex Medical Systems, Inc., Shirley, NY, USA). Each patient was seated upright, with straps stabilizing the chest, pelvis, and thigh of the currently examined limb. The hip position was set at 95°, while the knee starting position was at 90° flexion (Figure 1).

Three repetitions were performed for each lower extremity under isokinetic (for angular velocities of 90°/s and 150°/s) and isometric (for joint angles of 75° and 90°) conditions. Three training attempts preceded each trial. Based on the measurements, biomechanical parameters were determined, which are presented in Table 1.

**Figure 2:**
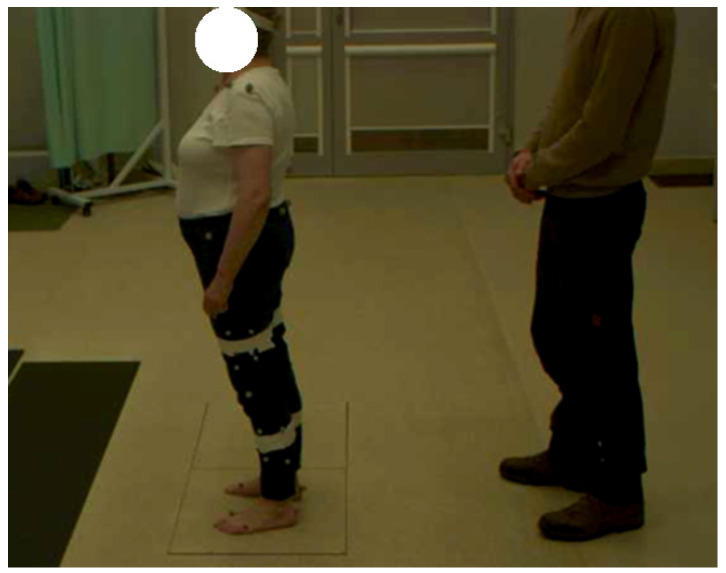
A patient undergoing assessment of changes in the COP position during tests with eyes closed or opened using a Kistler force plate and Qualisys software.

Changes in the COP position were recorded using a Kistler 600 × 500 mm, 9260AA6 force plate (Kistler Holding AG, Winterthur, Switzerland) during three 30 s repetitions performed while standing with eyes closed or opened. Data were collected with Qualisys ver. 2022.2 software (Qualisys AB, Göteborg, Sweden) integrated with a time-synchronized Kistler force plate (Figure 2) and averaged for all repetitions, individually for tests with eyes closed or opened.

**Figure 3:**
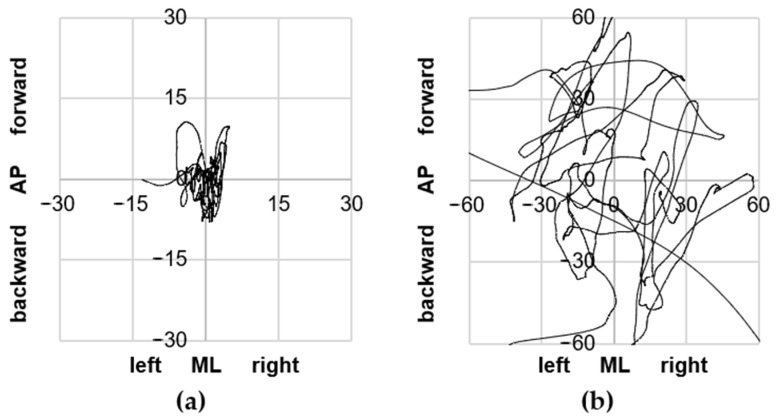
COP path [mm]—exemplary results obtained during tests with eyes closed for (**a**) a person from the control group and (**b**) a person from the balance disorder group.

Examples of the results that were further processed to obtain biomechanical parameters are presented in Figure 3.

Biomechanical parameters were determined based on these measurements, which are presented in Table 2.

Statistical analysis was performed using Statistica (TIBCO Software) 13.3. The Shapiro–Wilk test was performed to evaluate the normality of the data. The equality of variances was assessed using Levene’s test. The differences in parameters between groups were evaluated using Student's *t*-test (in the case of normally distributed data) or the Mann–Whitney U test (in the case of data with a non-normal distribution).

Multiple regression analysis was performed to determine the potential significance of muscle torques of knee flexors and extensors developed in isometric and isokinetic conditions in assessing balance disorders in older adults (Table 3). Parameters of the COP path were treated as dependent values quantitatively expressing the balance parameters of the subjects (it was checked if considered independent variables have a significant influence on this parameter individually in each analyzed group).

## 3. Results

Table 4 presents the mean, minimum, and maximum values, as well as standard deviations, of all (general, clinical, and biomechanical) parameters for the control and balance disorder groups and the results of statistical analyses of the data and the differences between groups. The obtained results of the DHI (0.77 ± 1.97 for the control group and 32.23 ± 13.29 for the balance disorder group), GDS (2.54 ± 2.34 for the control group and 4.25 ± 3.58 for the balance disorder group), FES-I (7.81 ± 1.41 for the control group and 12.46 ± 5.13 for the balance disorder group), and POMA (27.58 ± 1.10 for the control group and 23.35 ± 5.76 for the balance disorder group) tests, as well as Diff SMM (2.14 ± 2.31 for the control group and 0.87 ± 2.26 for the balance disorder group), clearly distinguish the groups from each other.

Table 5 presents the results of multiple regression for each of the assumed models. It is visible that in the case of models 1b and 3b, constants describing muscle torques of knee extensors generated in isokinetic conditions significantly influence the values of the COP path obtained by the balance disorder group during both tests (with eyes opened and closed).

## 4. Discussion

As expected, the research confirmed significant differences between the people with balance disorders and the control group regarding parameters such as the mean results of the GDS, FES-I, and POMA. It confirms the results of other authors’ research that a worse emotional state, a greater risk of recurrent falls, and fear of another fall characterize people with dizziness, balance disorders, and a history of falls [51,52,53,54]. The relationship between balance disorders and depressive disorders may be bidirectional. Significant differences determined for the GDS emphasize the possibility of the development of depression along with the appearance of balance disorders or, conversely, the influence of depression on the appearance of balance disorders, which is confirmed by the research conducted by Wang et al. and Li et al. [55,56]. The studied groups differed significantly in terms of DHI scores, which is evident because this was the criterion for distinguishing them.

Some authors suggested that SMM could be a parameter distinguishing people with balance disorders from the reference group [57,58]. Although larger skeletal muscle mass could be expected to be a protective factor against balance disorders, helping maintain balance and preventing falls, interestingly, no statistical differences in SMM parameters were found between the groups. The latest research by Arai et al. also confirmed that SMM could not be a parameter distinguishing the group with balance disorders from the reference group [59]. The research presented in this paper shows that it is not the absolute mass of skeletal muscle (SMM), but rather the degree of its deficit/excess (calculated as the difference between the expected muscle mass assessed using bioimpedance for a given person and the actual muscle mass) may be an essential parameter in the diagnosis of the risk of balance disorders.

In the case of biomechanical parameters, a statistically significant difference between analyzed groups occurred for the ikin-flex-asym parameter, which suggests the potential influence of asymmetry between the muscle torques developed by left and right knee joint extensors in isokinetic conditions on balance disorders. Moreover, all determined parameters characterizing the change in the COP also presented statistically significant differences between both groups, which is widely confirmed in the literature, including in studies conducted, among others, by Hewson et al. and Wiśniowska-Szurlej et al. [60,61]. Additionally, Pizzigalli et al. indicated that the COP path length, the COP velocity, and the range of sway in the AP and ML directions are the variables that distinguish older adult fallers from non-fallers [62].

The results obtained for the defined multiple regression models confirm the observations of differences between both groups. Models 1b and 3b suggest the influence of the muscle torques of the knee extensors and the asymmetry between the muscle torques developed by the knee joint extensors of the left and right lower extremities (both parameters achieved in isokinetic conditions) on the length of the COP path. The muscle torques of knee joint extensors obtained in isokinetic conditions may be an important parameter from the point of view of diagnosing people with balance disorders.

## 5. Limitations of the Study and Future Directions

While the present study provides novel insights into the role of knee joint muscle torques in maintaining balance among older adults, several limitations should be acknowledged.

Firstly, the study sample was recruited from a limited geographic area, which may affect the generalisability of the findings. Future studies should include larger, more diverse populations to confirm these results across different demographics.

Secondly, while the biomechanical measurements were standardized and performed using validated equipment, factors such as participant fatigue, motivation, or comorbidities (e.g., osteoarthritis) could have influenced the muscle torque outputs or postural control parameters.

Thirdly, the assessment of psychological variables, such as fear of falling and depression, was based solely on questionnaire data. Including objective neurocognitive assessments or vestibular function tests could enrich the clinical profile and provide further insight into balance disorders.

Future research should explore intervention strategies targeting strength asymmetry and evaluate its impact on postural stability and fall risk. Additionally, integrating wearable sensor technologies may offer more comprehensive and continuous monitoring of balance and mobility in real-life conditions.

## 6. Conclusions

Balance disorders are associated with a worse emotional state, fear of falling, and a greater risk of falling. While absolute muscle mass does not differentiate between people with and without balance disorders, the predictive factor, in this case, was the difference between the expected and actual muscle mass, indicating a potential deficit of muscle mass in a given person.

Moreover, the obtained results proved that muscle torques of knee extensors could be used to diagnose older patients with balance disorders, inspiring new directions for rehabilitation.

## Figures and Tables

**Table 1:** Designated biomechanical parameters to describe muscle torques of knee flexors and extensors in isometric and isokinetic conditions.

Biomechanical Parameter	Description
Ikin-ext [Nm/kg]	The averaged value of max. muscle torques of knee extensors (from all repetitions) obtained in isokinetic conditions for angular velocities of 90°/s and 150°/s, normalized to the person’s weight.
Ikin-ext-asym	The ratio between the averaged values of muscle torques of the left and right knee extensors obtained in isokinetic conditions for angular velocities of 90°/s and 150°/s normalized to the person’s weight.
Imet-ext [Nm/kg]	The averaged values of max. muscle torques of knee extensors (from all repetitions) obtained in isometric conditions for joint angles of 75° and 90°, normalized to the person’s weight.
Imet-ext-asym	The ratio between the averaged values of muscle torques of the left and right knee extensors obtained in isometric conditions for joint angles of 75° and 90° normalized to the person’s weight.
Ikin-flex [Nm/kg]	The averaged value of muscle torques of knee flexors obtained in isokinetic conditions for angular velocities of 90°/s and 150°/s, normalized to the person’s weight.
Ikin-flex-asym	The ratio between the averaged values of muscle torques of the left and right knee flexors obtained in isokinetic conditions for angular velocities of 90°/s and 150°/s normalized to the person’s weight.
Imet-flex [Nm/kg]	The averaged values of muscle torques of knee flexors obtained in isometric conditions for joint angles of 75° and 90°, normalized to the person’s weight.
Imet-flex-asym	The ratio between the averaged values of muscle torques of the left and right knee flexors obtained in isometric conditions for joint angles of 75° and 90° normalized to the person’s weight.

Ikin—isokinetic, Imet—isometric, Ext—extensors, Flex—flexors, and Asym—asymmetry.

**Table 2:** Designated biomechanical parameters to describe COP displacement while standing with eyes opened and closed.

Biomechanical Parameter	Description
COP Amp ML—EO [mm/cm]	The averaged amplitude of COP displacement in the mediolateral direction while standing with eyes opened, normalized to the person’s body height.
COP Amp AP—EO [mm/cm]	The averaged amplitude of COP displacement in the anteroposterior direction while standing with eyes opened, normalized to the person’s body height.
COP path—EO [mm/cm]	The averaged path length of the COP while standing with eyes open, normalized to the person’s body height.
COP Amp ML—EC [mm/cm]	The averaged amplitude of the COP displacement in the mediolateral direction while standing with eyes closed, normalized to the person’s body height.
COP Amp AP—EC [mm/cm]	The averaged amplitude of COP displacement in the anteroposterior direction while standing with eyes closed, normalized to the person’s body height.
COP path—EC [mm/cm]	The averaged path length of the COP while standing with eyes closed, normalized to the person’s body height.

COP—centre of pressure, Amp—amplitude, ML—mediolateral direction, AP—anteroposterior direction, EO—eyes opened, and EC—eyes closed.

**Table 3:** Models assumed for multiple regression analyses.

Model No.	Group	Dependent Variable	Independent Variable
Model 1a	Control group (n = 26)	COP path—EO [mm/cm]	Ikin-ext [Nm/kg] Ikin-ext-asym Imet-ext [Nm/kg] Imet-ext-asym
Model 1b	Balance disorder group (n = 26)		
Model 2a	Control group (n = 26)	COP path—EO [mm/cm]	Ikin-flex [Nm/kg] Ikin-flex-asym Imet-flex [Nm/kg] Imet-flex-asym
Model 2b	Balance disorder group (n = 26)		
Model 3a	Control group (n = 26)	COP path—EC [mm/cm]	Ikin-ext [Nm/kg] Ikin-ext-asym Imet-ext [Nm/kg] Imet-ext-asym
Model 3b	Balance disorder group (n = 26)		
Model 4a	Control group (n = 26)	COP path—EC [mm/cm]	Ikin-flex [Nm/kg] Ikin-flex-asym Imet-flex [Nm/kg] Imet-flex-asym
Model 4b	Balance disorder group (n = 26)		

Model 1—the potential influence of knee extensors on the COP path during standing with eyes opened. Model 2—the potential influence of knee flexors on the COP path during standing with eyes opened. Model 3—the potential influence of knee extensors on the COP path during standing with eyes closed. Model 4—the potential influence of knee flexors on the COP path during standing with eyes closed.

**Table 4:** General, clinical, and biomechanical parameters of the control and balance disorder groups.

		Total (n = 52)					Control Group (n = 26)					Balance Disorder Group (n = 26)					Levene’s Test	Test-*t*	Mann–Whitney U test
		Mean	Min.	Max.	STD	S-W Test	Mean	Min.	Max.	STD	S-W Test	Mean	Min.	Max.	STD	S-W Test			
						*p*					*p*					*p*	*p*	*p*	*p*
General Parameters	Age [years]	67.58	55.00	82.00	7.30	0.28	67.77	55.00	78.00	5.76	0.55	67.38	55.00	82.00	8.68	0.57	0.08	0.85	
	Body weight [kg]	75.10	47.00	99.00	13.42	0.32	79.85	55.00	99.00	13.28	0.17	70.35	47.00	91.00	12.01	0.26	0.30	0.009	
	Body height [cm]	163.92	151.00	187.00	8.80	0.03	165.50	151.00	183.00	8.51	0.15	162.35	151.00	187.00	8.98	0.54	0.99	0.20	
	BMI [kg/m^2^]	27.77	20.00	37.00	3.88	0.09	29.00	22.00	37.00	3.79	0.11	26.54	20.00	35.00	3.64	0.003	0.74	0.02	
Clinical Parameters	DHI	17.50	0.00	58.00	19.34	<0.001	0.77	0.00	6.00	1.97	<0.001	34.23	10.00	58.00	13.29	<0.001	<0.001		<0.001
	FES-I	10.13	0.00	22.00	4.41	<0.001	7.81	7.00	12.00	1.41	<0.001	12.46	0.00	22.00	5.13	0.08	<0.001		<0.001
	GDS	3.36	0.00	14.00	3.09	<0.001	2.54	0.00	8.00	2.34	<0.001	4.25	0.00	14.00	3.58	0.22	0.20		0.04
	POMA	25.59	10.00	28.00	4.51	<0.001	27.58	23.00	28.00	1.10	<0.001	23.35	10.00	28.00	5.76	0.72	<0.001		0.002
	SMM [kg]	26.94	18.40	39.20	5.47	0.01	28.12	19.70	39.20	5.46	<0.001	25.72	18.40	38.50	5.30	0.02	0.61	0.12	
	Diff SMM	1.51	−2.80	6.40	2.35	0.36	2.14	−2.70	6.40	2.31	0.90	0.87	−2.80	4.40	2.26	0.06	0.87	0.04	
Biomechanical Parameters	Ikin-ext [Nm/kg]	109.34	41.07	194.41	34.61	0.88	108.77	41.07	171.65	32.72	0.92	109.91	47.43	194.41	37.06	0.002	0.63	0.91	
	Ikin-ext-asym	0.12	0.00	0.38	0.10	0.002	0.14	0.00	0.38	0.10	0.11	0.11	0.00	0.37	0.09	0.03	0.52		0.27
	Imet-ext [Nm/kg]	148.57	44.35	322.04	53.32	0.08	144.35	44.35	268.56	50.59	0.93	152.78	68.56	322.04	56.61	0.02	0.79	0.57	
	Imet-ext-asym	0.14	0.00	0.51	0.12	<0.001	0.12	0.00	0.46	0.11	<0.001	0.15	0.01	0.51	0.13	0.007	0.29		0.62
	Ikin-flex [Nm/kg]	53.87	19.35	102.25	17.67	0.10	54.07	19.35	85.57	15.90	0.69	53.66	27.94	102.25	19.60	0.10	0.47		0.60
	Ikin-flex-asym	0.13	0.00	0.52	0.11	<0.001	0.09	0.00	0.29	0.08	0.01	0.16	0.00	0.52	0.13	0.26	0.07		0.033
	Imet-flex [Nm/kg]	63.32	25.60	121.38	22.50	0.02	62.79	25.60	104.64	19.16	0.99	63.84	32.77	121.38	25.79	0.04	0.20		0.69
	Imet-flex-asym	0.14	0.00	0.64	0.11	<0.001	0.14	0.00	0.64	0.13	<0.001	0.15	0.00	0.38	0.09	0.63	0.21		0.22
	COP Amp ML—EO [mm/cm]	0.07	0.01	0.14	0.03	0.35	0.06	0.01	0.11	0.03	0.70	0.08	0.04	0.14	0.03	0.04	0.36	0.008	
	COP Amp AP—EO [mm/cm]	0.10	0.04	0.23	0.04	0.01	0.08	0.04	0.13	0.03	0.72	0.12	0.07	0.23	0.04	0.005	0.10		<0.001
	COP path—EO [mm/cm]	1.50	0.82	2.46	0.41	0.04	1.23	0.82	1.72	0.23	0.50	1.76	0.99	2.46	0.38	0.65	0.04	<0.001	
	COP Amp ML—EC [mm/cm]	0.07	0.02	0.20	0.04	0.002	0.05	0.02	0.12	0.03	0.03	0.09	0.03	0.20	0.04	0.26	0.30		0.002
	COP Amp AP—EC [mm/cm]	0.12	0.05	0.30	0.05	<0.001	0.09	0.05	0.17	0.03	0.04	0.15	0.07	0.30	0.05	0.03	0.03		<0.001
	COP path—EC [mm/cm]	1.79	0.80	3.65	0.58	0.04	1.46	0.80	2.17	0.36	0.82	2.12	1.07	3.65	0.58	0.63	0.04	<0.001	

BMI—Body Mass Index, Ikin—isokinetic, Imet—isometric, Ext—extensors, Flex—flexors, Asym—asymmetry, COP—centre of pressure, Amp—amplitude, ML—mediolateral direction, AP—anteroposterior direction, EO—eyes opened, and EC—eyes closed.

**Table 5:** Results of multiple regression for each of assumed models.

		Unstandardized Coefficients		Standardized Coefficients	*t*-Value	*p*
		B	STD	Beta		
Model 1a	Constant	1.302	0.173		7.521	<0.001
	Ikin-ext [Nm/kg]	0.001	0.003	0.081	0.215	0.83
	Ikin-ext-asym	0.387	0.434	0.168	0.891	0.38
	Imet-ext [Nm/kg]	−0.002	0.002	−0.405	−1.079	0.29
	Imet-ext-asym	0.667	0.383	0.327	1.739	0.10
Model 1b	Constant	1.727	0.289		5.971	<0.001
	Ikin-ext [Nm/kg]	0.007	0.005	0.703	1.470	0.047
	Ikin-ext-asym	0.941	0.934	0.224	1.007	0.03
	Imet-ext [Nm/kg]	−0.005	0.003	−0.761	−1.589	0.13
	Imet-ext-asym	−0.499	0.640	−0.173	−0.780	0.44
Model 2a	Constant	1.462	0.200		7.301	<0.001
	Ikin-flex [Nm/kg]	−0.005	0.006	−0.372	−0.838	0.41
	Ikin-flex-asym	0.067	0.690	0.022	0.096	0.92
	Imet-flex [Nm/kg]	0.001	0.005	0.054	0.122	0.904
	Imet-flex-asym	0.112	0.398	0.064	0.281	0.782
Model 2b	Constant	1.699	0.283		6.013	<0.001
	Ikin-flex [Nm/kg]	−0.008	0.011	−0.404	−0.711	0.49
	Ikin-flex-asym	−0.168	0.739	−0.056	−0.227	0.82
	Imet-flex [Nm/kg]	0.005	0.009	0.344	0.597	0.56
	Imet-flex-asym	1.278	1.071	0.303	1.193	0.25
Model 3a	Constant	0.269	5.284		5.284	<0.001
	Ikin-ext [Nm/kg]	0.004	0.649	0.248	0.649	0.52
	Ikin-ext-asym	0.674	0.545	0.104	0.545	0.59
	Imet-ext [Nm/kg]	0.003	−1.163	−0.442	−1.163	0.26
	Imet-ext-asym	0.595	2.051	0.391	2.051	0.05
Model 3b	Constant	1.847	0.398		4.642	<0.001
	Ikin-ext [Nm/kg]	0.014	0.007	0.879	2.024	0.046
	Ikin-ext-asym	2.810	1.286	0.442	2.185	0.04
	Imet-ext [Nm/kg]	−0.009	0.004	−0.893	−2.053	0.05
	Imet-ext-asym	−0.960	0.881	−0.220	−1.091	0.29
Model 4a	Constant	1.924	0.310		6.207	<0.001
	Ikin-flex [Nm/kg]	−0.002	0.010	−0.101	−0.226	0.82
	Ikin-flex-asym	−0.589	1.068	−0.129	−0.551	0.59
	Imet-flex [Nm/kg]	−0.004	0.008	−0.209	−0.467	0.65
	Imet-flex-asym	−0.268	0.616	−0.101	−0.435	0.67
Model 4b	Constant	1.824	0.425		4.290	<0.001
	Ikin-flex [Nm/kg]	−0.004	0.017	−0.149	−0.264	0.79
	Ikin-flex-asym	0.282	1.111	0.063	0.254	0.802
	Imet-flex [Nm/kg]	0.003	0.013	0.148	0.259	0.80
	Imet-flex-asym	1.827	1.611	0.287	1.134	0.27

## Data Availability

The original contributions presented in this study are included in the article. Further inquiries can be directed to the corresponding author.

## Abbreviations

The following abbreviations are used in this manuscript:

DHI	Dizziness Handicap Inventory
GDS	Geriatric Depression Scale
POMA	Performance-Oriented Mobility Assessment
FRT	Functional Reach Test
FES-I	Falls Efficacy Scale—International
SMM	Skeletal muscle mass
Diff SMM	The difference between the obtained SMM value and its reference value
COP	Centre of pressure
WHO	World Health Organization
Ikin	Isokinetic
Imet	Isometric
Ext	Extensors
Flex	Flexors
Asym	Asymmetry
Amp	Amplitude
ML	Mediolateral direction
AP	Anteroposterior direction
EO	Eyes opened
EC	Eyes closed
